# Lymph Node Ratio as a Risk Factor for Early Recurrence in Older Patients with Stage II/III Gastric Cancer: A Retrospective Study

**DOI:** 10.3390/jcm14186609

**Published:** 2025-09-19

**Authors:** Yun-Chen Tsai, Hsin-Chen Lin, Chiann-Yi Hsu, Feng-Hsu Wu, Shao-Ciao Luo, Yu-Hsuan Shih

**Affiliations:** 1 Department of Medical Education, Taichung Veterans General Hospital, Taichung 407219, Taiwan; pennyswim1230@gmail.com; 2 Division of Medical Oncology, Department of Oncology, Taichung Veterans General Hospital, 1650 Taiwan Boulevard Sect. 4, Taichung 407219, Taiwan; jawlin15@hotmail.com; 3 Department of Post-Baccalaureate Medicine, College of Medicine, National Chung Hsing University, Taichung 402202, Taiwan; 4 Biostatistics Group, Department of Medical Research, Taichung Veterans General Hospital, Taichung 407219, Taiwan; chiann@vghtc.gov.tw; 5 Department of Critical Care Medicine, Taichung Veterans General Hospital, Taichung 407219, Taiwan; b101091110@tmu.edu.tw; 6 Division of General Surgery, Department of Surgery, Taichung Veterans General Hospital, Taichung 407219, Taiwan; ytppytpp@hotmail.com; 7 Department of Nursing, HungKuang University, Taichung 433304, Taiwan; 8 Institute of Medicine, College of Medicine, Chung Shan Medical University, Taichung 402306, Taiwan

**Keywords:** gastric cancer, early recurrence, older patients, lymph node ratio, adjuvant chemotherapy

## Abstract

**Background/Objectives:** Older adults with gastric cancer often have poorer prognoses than younger patients. Early recurrence, within two years after curative surgery, is associated with poor outcomes, but its risk factors remain unclear. This study aimed to identify clinicopathological predictors of early recurrence in older patients with stage II/III disease. **Methods:** We retrospectively reviewed 604 patients with stage II/III gastric cancer who underwent curative surgery from 2009 to 2020. After exclusions, 237 patients aged ≥65 years were analyzed. Clinicopathological variables were compared between those with and without early recurrence, and risk factors were assessed using logistic regression. **Results:** Among the 237 patients studied, 103 had recurrence following surgery, of whom 72 (69.9%) were categorized as early recurrence. Distant metastasis was the most common pattern (59.7%), followed by peritoneal (45.8%) and locoregional (33.3%) recurrences. Multivariate analysis identified a lymph node ratio (LNR) > 0.17 as an independent risk factor for early recurrence (odds ratio (OR), 5.30; 95% confidence interval (CI), 2.07–13.53; *p* < 0.001). **Conclusions:** Early recurrence is frequent among older patients with stage II/III gastric cancer, with distant metastasis as the predominant pattern. An LNR > 0.17 predicts higher recurrence risk. While adjuvant chemotherapy showed a trend toward reduced risk, statistical significance was not reached. Further prospective studies are necessary to confirm these findings.

## 1. Introduction

Gastric cancer remains a major global health challenge, ranking as the fifth most commonly diagnosed cancer and the fifth leading cause of cancer-related mortality worldwide [1]. Its incidence is particularly high in Asian countries, where it is among the top ten causes of cancer-related deaths in Taiwan. Gastric cancer is widely considered an age-related disease, with over 60% of patients diagnosed at 65 years or older [2,3,4]. Older patients often present with more comorbidities and are at a higher risk of treatment-related complications. Several studies have demonstrated that age is an important adverse prognostic factor for survival following gastric surgery [5].

For patients with stage II and III gastric cancer, the standard treatment consists of surgical resection with regional lymphadenectomy, followed by adjuvant chemotherapy [6,7]. However, recurrence remains common, affecting between one-third and two-thirds of patients after surgery [8,9]. Postoperative recurrence is frequently associated with poor outcomes, with various recurrence patterns reported. While the proportions of distant metastasis, locoregional recurrence, and peritoneal metastasis differ across studies, most indicate that distant metastasis is the most common pattern. Most recurrences occur within the first 2 years, and patients experiencing early recurrence during this period tend to have a particularly poor prognosis and high mortality rates [10,11].

Given these findings, both older age and early recurrence are linked to poor outcomes in patients with gastric cancer. However, limited studies have explored the patterns and risk factors of early recurrence, specifically in older patients with gastric cancer. This single-institution study focused on examining the patterns of early recurrence and identifying its contributing factors in older patients with stage II/III gastric cancer following curative gastrectomy, providing insights for postoperative follow-up in this high-risk group.

## 2. Methods

### 2.1. Patients

**Figure 1:**
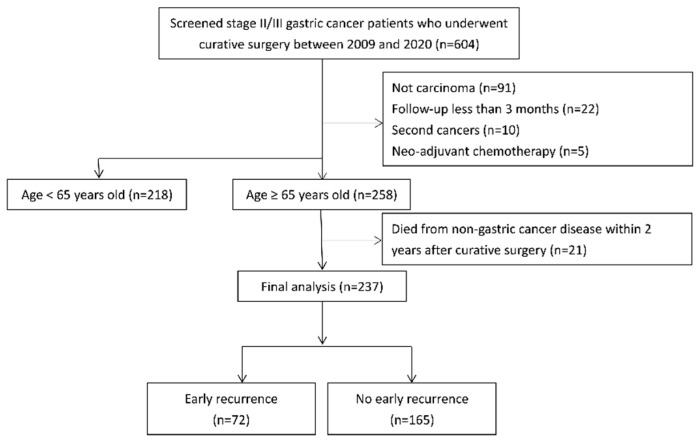
Flowchart of patient selection.

We conducted a retrospective analysis of medical records from Taichung Veterans General Hospital between January 2009 and December 2020. The study population consisted of patients aged 20 years or older with pathologically confirmed stage II/III gastric carcinoma who underwent curative surgery with D1 or D2 lymphadenectomy. Initially, 604 consecutive patients were assessed. We excluded 91 patients with non-carcinoma histology, 22 with a follow-up period of less than 3 months, 10 diagnosed with another active cancer, and five who received neoadjuvant chemotherapy. Among the 258 patients aged 65 years or older, 21 who died from non-gastric cancer-related causes within 2 years after surgery were excluded to focus the analysis solely on factors related to the early recurrence of gastric cancer. This resulted in a final cohort of 237 patients for analysis, among whom 72 experienced early recurrence within 2 years after surgery, while 165 did not (Figure 1). Postoperative follow-up strategies included history-taking and physical examinations conducted every three to six months for up to five years, abdominal computed tomography scans at corresponding intervals, and esophagogastroduodenoscopy when clinically indicated. Patients who received at least 8 weeks of postoperative chemotherapy were classified as having received adjuvant chemotherapy. Chemotherapy regimens were determined by the treating physicians and included fluoropyrimidines such as S-1, capecitabine, or 5-fluorouracil, administered either as monotherapy or in combination with platinum or taxane agents. Details of chemotherapy regimens are summarized in Table S1. This study was conducted in compliance with the principles of the Declaration of Helsinki. The institutional review board of Taichung Veterans General Hospital approved the study protocol and granted a waiver for informed consent due to its retrospective design (Approval No. CE24060C; approval date 23 February 2024). The reporting of this study conforms to the Strengthening the Reporting of Observational Studies in Epidemiology (STROBE) guidelines [12].

### 2.2. Variables and Outcome Measurements

The clinical and pathological information of the patients was collected retrospectively. Cancer staging was classified according to the eighth edition of the American Joint Committee on Cancer (AJCC) staging system. To account for the impact of age on comorbidities, the age-adjusted Charlson Comorbidity Index (aCCI) was used. ECOG performance status was dichotomized into 0–1 versus >1 to reflect good versus poor functional status. Patients were classified as having early recurrence if tumor recurrence occurred within 2 years after surgery. Recurrence types were categorized as locoregional recurrence (including local lymph node metastasis), peritoneal recurrence, and distant metastasis.

The lymph node ratio (LNR) is defined as the proportion of metastatic lymph nodes to the total number of lymph nodes harvested during surgery [13]. Based on the receiver operating characteristic (ROC) curve analysis, we identified an optimal cut-off value of 0.17 for predicting early recurrence, with an area under the ROC curve (AUC) of 0.758.

Several hematological parameters were calculated to evaluate inflammatory and nutritional status, including the neutrophil-to-lymphocyte ratio (NLR), platelet-to-lymphocyte ratio (PLR), lymphocyte-to-monocyte ratio (LMR), prognostic nutritional index (PNI), and pan-immune inflammation value (PIV). All hematological parameters, including NLR, PLR, LMR, PIV, and PNI, were calculated based on preoperative blood samples collected within two weeks prior to surgery.

NLR is defined as the ratio of the absolute neutrophil count to the absolute lymphocyte count. PLR is the ratio of the absolute platelet count to the absolute lymphocyte count [14,15]. LMR represents the ratio of the absolute lymphocyte count to the absolute monocyte count [16]. PIV, an indicator of overall inflammatory burden, is calculated using the formula: (neutrophil count × platelet count × monocyte count)/lymphocyte count, with all values measured in 10^9^/L [17]. These ratios reflect various aspects of the systemic inflammatory response. PNI, a measure of nutritional and immunological status, is calculated as (serum albumin in g/dL × 10) + (5 × lymphocyte count in 10^9^/L) [18]. Since the AUC for predicting early recurrence was less than 0.6 for these parameters, making it difficult to determine an optimal cut-off value, we used the median values of the NLR, PLR, LMR, PIV, and PNI as cut-off values.

### 2.3. Statistical Analyses

The clinicopathological features of patients were analyzed and compared between the early recurrence and no early recurrence groups. Continuous variables were presented as medians with interquartile ranges, while categorical variables were reported as frequencies and percentages. Comparisons between groups were performed using the Mann–Whitney U test for continuous data and either the chi-square test or Fisher’s exact test for categorical data, depending on applicability. Survival curves were generated using the Kaplan–Meier method and compared with the log-rank test. Logistic regression analysis was employed to identify risk factors for early recurrence, with results reported as odds ratios (ORs) and 95% confidence intervals (CIs). Univariate analysis was performed to evaluate potential risk factors for early recurrence, and variables with a *p*-value < 0.1 were further analyzed in multivariate logistic regression models to identify independent predictors. A *p*-value < 0.05 was considered statistically significant. All statistical analyses were conducted using the Statistical Package for the Social Sciences (IBM SPSS version 22.0; International Business Machines Corp, New York, NY, USA).

## 3. Results

### 3.1. Recurrence Timing and Early Recurrence Pattern

**Figure 2:**
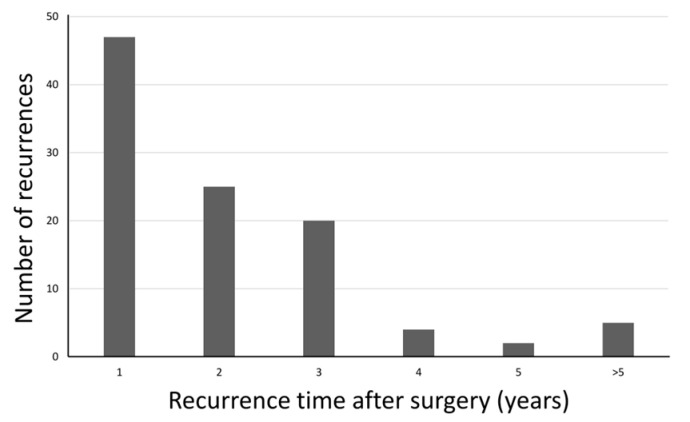
Timing of Recurrence After Surgery. Distribution of recurrence times post-surgery among 103 patients with recurrence. Early recurrence (within 2 years) accounted for 69.9% of cases.

**Figure 3:**
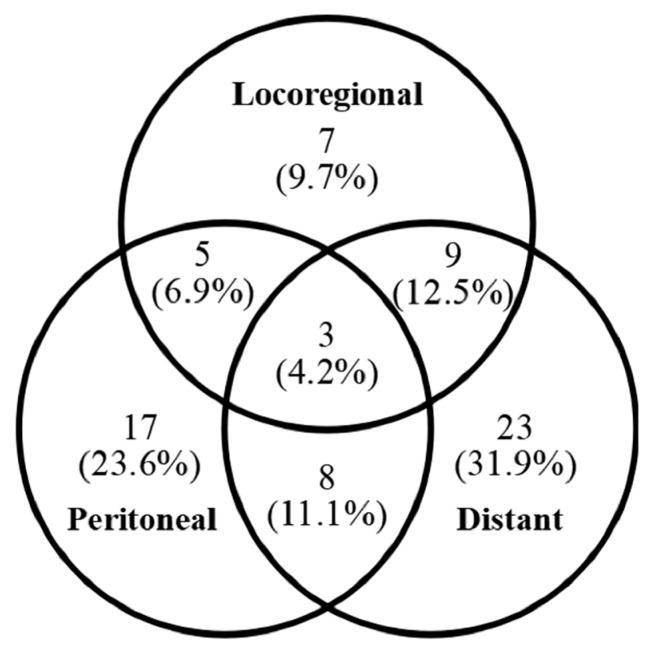
Patterns of Early Recurrence. Patterns of early recurrence in 72 patients who experienced recurrence within the first 2 years. Distant metastasis (59.7%) was the most frequent, followed by peritoneal (45.8%) and locoregional recurrence (33.3%).

**Figure 4:**
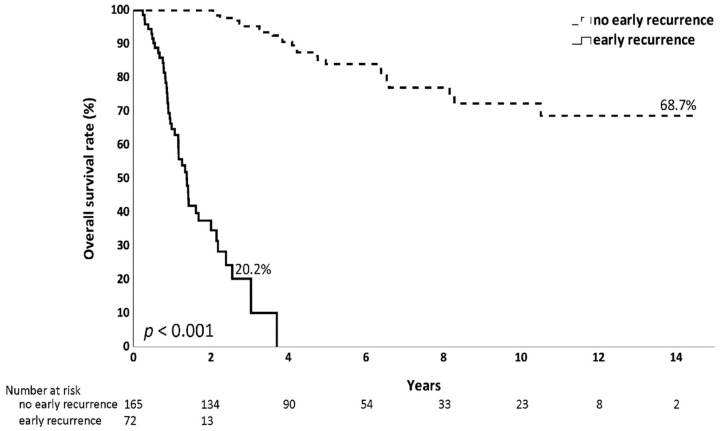
Overall survival of patients aged 65 years or older with stage II/III resected gastric cancers, early recurrence or not. Kaplan–Meier curves show significantly lower overall survival in patients with early recurrence compared to those without early recurrence.

Among the 237 analyzed patients, 103 experienced recurrence after surgery. Of these, 72 patients (69.9%) had a recurrence within the first 2 years (Figure 2). Regarding the pattern of early recurrence in these 72 patients, 43 (59.7%) developed distant metastasis, 33 (45.8%) had peritoneal metastasis and 24 (33.3%) experienced locoregional recurrence (Figure 3). The recurrence predominantly presented as a hematogenous spread or peritoneal dissemination. The prognosis was notably poor for older patients who experienced early recurrence after surgery, with a 3-year overall survival rate of only 20.2%, in contrast to a 68.7% long-term survival rate in patients without early recurrence (Figure 4).

### 3.2. Comparison of the Clinical Characteristics Between the Early Recurrence and No Early Recurrence Groups

Patients with early recurrence demonstrated distinct clinical features compared to those without early recurrence (Table 1). A greater proportion of patients in the early recurrence group had an ECOG performance status > 1 (47.2% vs. 33.9%, *p* = 0.053) and were more likely to have undergone total gastrectomy (48.6% vs. 29.1%, *p* = 0.004). The tumor size was significantly larger in the early recurrence group, with 76.4% of patients presenting with tumors > 4 cm, compared to 50.9% in the no early recurrence group (*p* < 0.001). Advanced-stage disease (Stage III) was more prevalent in the early recurrence group (79.2% vs. 46.1%, *p* < 0.001), as were lymphovascular invasion (87.5% vs. 71.3%, *p* = 0.007) and positive surgical margins (12.5% vs. 3.0%, *p* = 0.013).

Among the 237 patients analyzed, the median number of dissected lymph nodes was 31, with approximately 87.3% (207 patients) having ≥15 lymph nodes dissected. A total of 165 patients (69.6%) underwent D2 dissection. A Kaplan–Meier analysis comparing DFS and OS between patients who underwent D1 versus D2 dissection is presented in Figure S1. The early recurrence group exhibited significantly higher N stages, with 76.4% classified as N2–3 compared with 48.5% in the no early recurrence group (*p* < 0.001). A greater proportion had an LNR > 0.17 (66.7% vs. 24.2%, *p* < 0.001). Additionally, elevated levels of carcinoembryonic antigen (CEA) and carbohydrate antigen 19-9 (CA19-9) were more frequently observed in the early recurrence group (33.3% vs. 11.5%, *p* < 0.001; 36.8% vs. 16.0%, *p* = 0.002, respectively). Adjuvant chemotherapy was administered to 169 patients (71.3%). Among patients with ECOG performance status of 0–1, 121 of 147 (81.8%) received adjuvant chemotherapy, whereas 48 of 90 patients (53.3%) with ECOG > 1 received chemotherapy. Hematologic adverse events related to adjuvant chemotherapy are summarized in Table S2.

### 3.3. Risk Factors for Early Recurrence

Univariate and multivariate analyses were conducted to identify the risk factors for early recurrence in older patients with gastric cancer (Table 2). In the univariate analysis, several factors were found to be significantly associated with early recurrence. These included tumor size greater than 4 cm (OR, 3.12; 95% CI: 1.67–5.82; *p* < 0.001), total gastrectomy (OR, 2.31; 95% CI: 1.30–4.08; *p* = 0.004), ECOG performance status > 1 (OR, 1.74; 95% CI: 0.99–3.06; *p* = 0.054), BMI ≥ 24 (OR, 0.36; 95% CI: 0.13–0.98; *p* = 0.045), N2–3 stage (OR, 3.44; 95% CI: 1.84–6.41; *p* < 0.001), LNR > 0.17 (OR, 6.25; 95% CI: 3.41–11.45; *p* < 0.001), lymphovascular invasion (OR, 2.81; 95% CI: 1.29–6.11; *p* = 0.009), perineural invasion (OR, 2.22; 95% CI: 1.23–3.99; *p* = 0.008), positive surgical margins (OR, 4.57; 95% CI: 1.47–14.17; *p* = 0.008), elevated CEA levels (OR, 3.86; 95% CI: 1.92–7.78; *p* < 0.001), and elevated CA19-9 levels (OR, 3.06; 95% CI: 1.50–6.23; *p* = 0.002). Adjuvant chemotherapy was associated with a reduced risk of early recurrence (OR, 0.55; 95% CI: 0.30–1.00; *p* = 0.049).

In the multivariate analysis, an LNR > 0.17 remained the only independent risk factor significantly associated with early recurrence (OR, 5.30; 95% CI: 2.07–13.53; *p* < 0.001). Although adjuvant chemotherapy did not reach statistical significance, it showed a trend toward reducing the risk of early recurrence (OR, 0.43; 95% CI: 0.16–1.12; *p* = 0.08). Other variables, such as ECOG performance status, BMI, N stage, tumor size, total gastrectomy, lymphovascular invasion, perineural invasion, and elevated tumor markers, were included in the model based on a univariate *p*-value less than 0.1 but were not statistically significant in the multivariate analysis.

## 4. Discussion

This study assessed patients aged 65 years or older with stage II/III gastric cancer who underwent curative surgery. We observed that most recurrences occurred within the first 2 years. Distant metastasis was the most common pattern of recurrence, followed by peritoneal metastasis and locoregional recurrence. An LNR > 0.17 was associated with a higher risk of early recurrence.

Several retrospective studies have examined the timing and patterns of gastric cancer recurrence. For example, Kang et al. [19] analyzed 417 patients who underwent gastric resection and reported that 194 experienced recurrence, with 129 cases (66.5%) occurring within the first 2 years post-surgery. Among these early recurrences, 43.4% were hematogenous, 52.7% were locoregional, and 45.7% were peritoneal [19]. Similarly, Liu et al. [10] studied 1304 patients who underwent curative resection and found that 554 cases of recurrence occurred within 2 years, accounting for 69.9% of the 793 total recurrences observed. Of these, approximately 32.4% were locoregional, 44.3% involved distant metastasis, and 13.7% involved peritoneal implants [10]. Another study reviewing 776 patients reported recurrence patterns in 300 cases, identifying 51 peritoneal, 176 locoregional, and 164 distant recurrences [11].

In our study, which focused on patients aged 65 years or older with stage II/III gastric cancer who underwent curative resection, approximately 69.9% of the 103 patients who experienced recurrence did so within the first 2 years. In this high-risk cohort, distant metastasis was observed in approximately 59.7% of the cases, and peritoneal metastasis was observed in 45.8%, indicating a higher prevalence of distant and peritoneal metastases compared to previous studies. Our findings align with those of earlier reports that indicated that recurrence patterns vary by age. For example, Qiu et al. [20] noted that younger patients (≤45 years) exhibited higher rates of peritoneal recurrence, with two distinct peaks at 8.5 and 41.5 months post-surgery. In contrast, older patients experienced earlier and higher rates of distant recurrence, with a peak at 6 months [20]. These findings underscore the importance of vigilant and comprehensive postoperative follow-up, particularly for high-risk patients such as those in our study cohort.

Several risk factors for early recurrence in patients with gastric cancer have been identified in previous studies. Kang et al. reported that age, pathological T stage, pathological N stage, Lauren histotype, and lymphovascular invasion were significant risk factors for early recurrence in patients with pT2-4a stage gastric cancer [19]. Another study demonstrated that pathological lymph node metastasis ≥ 14, preoperative CA19-9 levels ≥37 IU/mL, and intraoperative blood loss ≥ 445 mL were independent risk factors for early recurrence after curative gastrectomy in patients with stage III gastric cancer [21]. A multicenter retrospective study by Yagi et al. further highlighted that elevated CEA levels and NLR were associated with early recurrence following radical gastrectomy and adjuvant chemotherapy in patients with stage II/III gastric cancer [22]. In a large-scale study involving 1511 patients, additional risk factors for early recurrence were identified, including tumor location, a positive lymph node ratio ≥ 0.335, pTNM stage III, lymphocyte count < 1.5 × 10^9^/L, postoperative infectious complications, and undergoing fewer than six cycles of adjuvant chemotherapy. Based on these factors, a nomogram was developed to predict early recurrence risk in patients with stage II/III gastric cancer, demonstrating high accuracy and clinical utility [23].

Our study identified an LNR > 0.17 as a significant risk factor for early recurrence in patients aged 65 years or older with stage II/III gastric cancer who underwent curative surgery. LNR has increasingly been recognized as a critical prognostic indicator for patients with gastric cancer following curative resection. Numerous studies have consistently shown that LNR is a strong predictor of recurrence and overall survival, often outperforming traditional N staging in prognostic accuracy [24,25]. While specific LNR cut-offs vary across studies, higher LNR values are generally associated with poorer overall survival and disease-free survival. The LNR cut-off identified in our study (0.17) differs from that reported in other cohorts, such as Min Man et al. [23], who found a threshold of 0.335. While the median number of lymph nodes dissected in our cohort was relatively high (31 nodes), and 87.3% of patients had ≥15 lymph nodes retrieved, differences in surgical practice, pathology processing, and patient characteristics may account for the variation. Therefore, our findings regarding the prognostic value of LNR should be interpreted with caution and validated in independent cohorts.

Komatsu et al. identified the optimal cut-off for stratifying prognosis as 0.2, whereas Aoyama et al. used 0.23, and Ke et al. employed cut-offs of 0.25 and 0.50 [26,27,28,29]. This metric is particularly relevant for older patients, who are more likely to undergo limited lymph node dissection due to increased surgical risks. Additionally, LNR is less influenced by the total number of lymph nodes retrieved, making it less susceptible to stage migration compared to the conventional TNM staging system [30]. These findings highlight the potential of LNR as a complementary or alternative tool to traditional staging systems. By integrating LNR into postoperative risk stratification, clinicians may better tailor follow-up strategies and surveillance intensity for patients with gastric cancer, particularly those in high-risk groups.

This study included patients who underwent both D1 and D2 dissections, rather than restricting the analysis to D2 dissections alone. This decision reflects the ongoing controversy regarding the benefits of D2 lymph node dissection in older patients with gastric cancer. Some studies suggest that D2 dissection does not improve survival and may increase complications in older patients [31,32]. However, the Italian Gastric Cancer Study Group’s 15-year follow-up demonstrated improved disease-specific survival with D2 dissection in advanced cases, while better outcomes were observed with D1 dissection in patients over 70 years old and those with early-stage disease [33]. In our cohort, approximately 70% of patients aged over 65 underwent standard D2 dissection. Patients who underwent < D2 dissections were not excluded to better reflect real-world practice. A key advantage of using the LNR is its ability to account for variations in the number of dissected lymph nodes. Since not all older patients can undergo complete D2 dissection in clinical settings, the finding that LNR is associated with early recurrence retains scientific significance and provides valuable insights into this high-risk population.

Although adjuvant chemotherapy is the standard of care for stage II/III gastric cancer, its benefits for older patients remain inconclusive. Older patients often face unique challenges, including shorter life expectancy, a higher prevalence of comorbidities, and an increased risk of treatment-related complications. Recent retrospective studies suggest that adjuvant chemotherapy may improve disease-free and overall survival in older patients with stage II/III gastric cancer compared to those who do not receive treatment [34]. However, older patients are significantly less likely to undergo adjuvant chemotherapy due to various barriers [35]. These barriers include age-related physiological changes, comorbidities, and frailty, all of which may reduce treatment tolerance and efficacy. Despite the challenges of delivering full-course adjuvant chemotherapy in older patients, previous studies have demonstrated that even incomplete treatment may confer recurrence-reducing benefits. In our study, adjuvant chemotherapy was associated with a trend toward reduced early recurrence (*p* = 0.08), though this potential benefit should be interpreted with caution. Furthermore, prior studies have reported increased short-term mortality in patients aged ≥76 years, highlighting the need for age-specific prognostic tools and individualized treatment approaches [5]. As our study lacked comprehensive data on non-hematologic toxicities and treatment adherence. These factors are particularly relevant in the older population and may significantly influence the real-world effectiveness of chemotherapy.

To address these challenges, comprehensive geriatric assessments are recommended to guide personalized treatment decisions. Tailored approaches, such as dose reductions or monotherapy for frail patients, may help optimize the balance between efficacy and toxicity [36]. While our findings suggest a potential role for adjuvant chemotherapy in selected older patients, the lack of statistical significance and missing tolerability data underscore the need for further prospective studies to confirm its real-world effectiveness.

Several studies have explored the prognostic value of inflammatory biomarkers in resectable gastric cancer. Elevated preoperative NLR and PLR are associated with poorer overall survival and disease-free survival, as well as advanced tumor stages and unfavorable clinicopathological features [37,38,39]. Similarly, the PNI has been identified as a significant predictor of survival and postoperative complications in patients with gastric cancer. Studies have consistently reported that a low PNI is correlated with worse overall and disease-free survival [40,41,42]. However, limited research has examined the impact of inflammatory markers and PNI on early recurrence in older patients. While recent findings highlight the negative prognostic implications of inflammatory biomarkers and nutritional indices in patients with resectable gastric cancer, our study found no significant association between these biomarkers and early recurrence in those aged 65 years and older. It is important to note that age itself may influence baseline levels of these biomarkers. A growing body of evidence has shown that older adults tend to exhibit higher values of NLR and PLR and lower values of LMR and PNI, even in the absence of active disease or comorbidities. This phenomenon, known as “inflammaging,” reflects age-related immune dysregulation and nutritional decline, and may confound the prognostic utility of these markers in geriatric populations. Therefore, caution is warranted when interpreting these indices in older patients [43,44,45]. Further research is needed to clarify the role of these factors in this high-risk population.

Our study had several limitations. First, being a retrospective analysis conducted at a single institution with a relatively small patient cohort, it was prone to selection bias, potentially limiting the generalizability of the findings to broader populations. Second, the retrospective design lacked certain key information relevant to older adults, such as comprehensive geriatric assessments, quality-of-life measurements, and major postoperative complications like anastomotic leakage or infection. These unmeasured factors could have impacted both short- and long-term outcomes and limited our ability to adjust for these risks in the analysis.

Third, to better reflect real-world practice, we included patients who underwent both D1 and D2 dissections, which inevitably increased the heterogeneity of the study cohort. Although stratified survival analyses were performed to compare D1 versus D2, residual confounding cannot be excluded.

Fourth, data on surgeon volume, center procedural volume, pathology specimen handling, and strict adherence to standardized imaging follow-up protocols were not available. These unmeasured factors may introduce performance and detection biases, and it is possible that the prognostic value of LNR partly reflects variability in quality of care rather than purely tumor biology.

Fifth, detailed information on chemotherapy treatment exposure, including relative dose intensity (RDI), the number of administered cycles, and time to treatment initiation (TTI), was not consistently available for all patients. Although we summarized the chemotherapy regimens in Table S1 to enhance transparency, the absence of these metrics limits our ability to fully account for treatment intensity and its effects.

Moreover, we reported hematologic adverse events (Table S2), while non-hematologic toxicities—such as gastrointestinal or neurotoxic side effects—were not systematically collected. Data on chemotherapy adherence or completion were also unavailable. These missing elements restrict our capacity to evaluate the real-world tolerability of adjuvant chemotherapy in older adults. As such, we advise cautious interpretation of the observed protective association between chemotherapy and early recurrence, especially in a vulnerable elderly population where toxicity burden and treatment adherence may vary substantially in clinical practice.

Sixth, the optimal cut-off value for LNR was derived from ROC analysis within the same dataset. Without internal validation using bootstrap-based methods or external validation in an independent cohort, this may increase the risk of overfitting and optimism bias. We therefore caution against overinterpretation of this threshold and have avoided making strong clinical recommendations based solely on this value.

Seventh, we categorized inflammatory and nutritional biomarkers based on the median due to limited discriminatory ability (low AUC). We acknowledge that this approach may introduce misclassification and reduce the power to detect non-linear relationships. Continuous modeling strategies, such as splines, could have provided a more nuanced assessment but were not feasible in this study.

Finally, the limited sample size, particularly within the early recurrence group, may have reduced the statistical power to detect weaker associations or interactions among risk factors. Future studies should include larger, multicenter cohorts with prospective designs to validate our findings. Incorporating more refined cut-off values for inflammatory and nutritional biomarkers, as well as integrating comprehensive geriatric assessments, may enhance the accuracy and clinical applicability of such models.

## 5. Conclusions

This study highlights that early recurrence, occurring within two years post-surgery, is frequent among older patients with stage II/III gastric cancer, with distant metastasis as the most common recurrence type. An LNR exceeding 0.17 was identified as a significant risk factor for early recurrence. While adjuvant chemotherapy showed a trend toward reduced risk, statistical significance was not reached. Prospective research is necessary to verify these results and refine postoperative management strategies.

## Figures and Tables

**Table 1:** Patient characteristics stratified according to early recurrence or not.

	Total (n = 237)		No Early Recurrence (n = 165)		Early Recurrence (n = 72)		*p*-Value
**Age (years), median (IQR)**	75.0	(70–81)	74.0	(70–81)	77.0	(71–83)	0.256
**Sex, n (%)**							0.418
Female	78	(32.9%)	57	(34.5%)	21	(29.2%)	
Male	159	(67.1%)	108	(65.5%)	51	(70.8%)	
**BMI, n (%)**							0.096
<18.5	19	(8.0%)	10	(6.1%)	9	(12.5%)	
18.5–24	119	(50.2%)	80	(48.5%)	39	(54.2%)	
≥24	99	(41.8%)	75	(45.5%)	24	(33.3%)	
**ECOG Performance Status, n (%)**							0.053
0–1	147	(62.0%)	109	(66.1%)	38	(52.8%)	
>1	90	(38.0%)	56	(33.9%)	34	(47.2%)	
**aCCI, n (%)**							0.341
0–3	83	(35.0%)	61	(37.0%)	22	(30.6%)	
>3	154	(65.0%)	104	(63.0%)	50	(69.4%)	
**Location, n (%)**							0.917
Proximal	57	(24.1%)	40	(24.2%)	17	(23.6%)	
Non-proximal	180	(75.9%)	125	(75.8%)	55	(76.4%)	
**Size, n (%)**							<0.001
≤4	98	(41.4%)	81	(49.0%)	17	(23.6%)	
>4	139	(58.6%)	84	(50.9%)	55	(76.4%)	
**Differentiation, n (%)**							0.207
Well to moderate	37	(15.6%)	29	(17.6%)	8	(11.1%)	
Poor differentiation	200	(84.4%)	136	(82.4%)	64	(88.9%)	
***Helicobacter pylori* infection, n/total n (%)**							0.979
No	180/219	(82.2%)	125/152	(82.2%)	55/67	(82.1%)	
Yes	39/219	(17.8%)	27/152	(17.8%)	12/67	(17.9%)	
**Signet ring feature, n/total n (%)**							0.367
No	170/236	(72.0%)	121/164	(73.8%)	49/72	(68.1%)	
Yes	66/236	(28.0%)	43/164	(26.2%)	23/72	(31.9%)	
**Gastrectomy type, n (%)**							0.004
Subtotal gastrectomy	154	(65.0%)	117	(70.9%)	37	(51.4%)	
Total gastrectomy	83	(35.0%)	48	(29.1%)	35	(48.6%)	
**Lymphadenectomy type, n (%)**							0.205
<D2 dissection	72	(30.4%)	46	(27.9%)	26	(36.1%)	
D2 dissection	165	(69.6%)	119	(72.1%)	46	(63.9%)	
**Stage, n (%)**							<0.001
Stage II	104	(43.9%)	89	(53.9%)	15	(20.8%)	
Stage III	133	(56.1%)	76	(46.1%)	57	(79.2%)	
**T Stage, n (%)**							0.148
T 1–2	35	(14.8%)	28	(17.0%)	7	(9.7%)	
T 3–4	202	(85.2%)	137	(83.0%)	65	(90.3%)	
**N Stage, n (%)**							<0.001
N 0–1	102	(43.0%)	85	(51.5%)	17	(23.6%)	
N 2–3	135	(57.0%)	80	(48.5%)	55	(76.4%)	
**Lymph nodes ratio, n (%)**							<0.001
≤0.17	149	(62.9%)	125	(75.8%)	24	(33.3%)	
>0.17	88	(37.1%)	40	(24.2%)	48	(66.7%)	
**Lymphovascular invasion, n/total n (%)**							0.007
No	56/236	(23.7%)	47/164	(28.7%)	9/72	(12.5%)	
Yes	180/236	(76.3%)	117/164	(71.3%)	63/72	(87.5%)	
**Perineural invasion, n/total n (%)**							0.007
No	102/234	(43.6%)	80/162	(49.4%)	22/72	(30.6%)	
Yes	132/234	(56.4%)	82/162	(50.6%)	50/72	(69.4%)	
**Margin, n (%)**							0.013
Negative	223	(94.1%)	160	(97.0%)	63	(87.5%)	
Positive	14	(5.9%)	5	(3.0%)	9	(12.5%)	
**Adjuvant chemotherapy, n (%)**							0.048
No	68	(28.7%)	41	(24.8%)	27	(37.5%)	
Yes	169	(71.3%)	124	(75.2%)	45	(62.5%)	
**CEA level, n/total n (%)**							<0.001
Normal	185/226	(81.9%)	139/157	(88.5%)	46/69	(66.7%)	
Elevated (≥5 U/mL)	41/226	(18.1%)	18/157	(11.5%)	23/69	(33.3%)	
**CA19-9 level, n/total n (%)**							0.002
Normal	146/188	(77.7%)	110/131	(84.0%)	36/57	(63.2%)	
Elevated (≥34 U/mL)	42/188	(22.3%)	21/131	(16.0%)	21/57	(36.8%)	
**Neutrophil-to-lymphocyte ratio, n/total n (%)**							0.113
≤2.7	120/233	(51.5%)	89/162	(54.9%)	31/71	(43.7%)	
>2.7	113/233	(48.5%)	73/162	(45.1%)	40/71	(56.3%)	
**Platelet-to-lymphocyte ratio, n/total n (%)**							0.341
≤164	116/233	(49.8%)	84/162	(51.9%)	32/71	(45.1%)	
>164	117/233	(50.2%)	78/162	(48.1%)	39/71	(54.9%)	
**Lymphocyte-to-monocyte ratio, n/total n (%)**							0.137
≤3.1	124/233	(53.2%)	81/162	(50.0%)	43/71	(60.6%)	
>3.1	109/233	(46.8%)	81/162	(50.0%)	28/71	(39.4%)	
**Prognostic Nutritional index, n (%)**							0.717
≤45	126	(53.2%)	89	(53.9%)	37	(51.4%)	
>45	111	(46.8%)	76	(46.1%)	35	(48.6%)	
**Pan-immune inflammation value, n/total n (%)**							0.341
≤305	116/233	(49.8%)	84/162	(51.9%)	32/71	(45.1%)	
>305	117/233	(50.2%)	78/162	(48.1%)	39/71	(54.9%)	

Percentages are based on non-missing data for each variable. aCCI, age-adjusted Charlson comorbidity index; BMI, body mass index; ECOG, Eastern Cooperative Oncology Group; CEA, carcinoembryonic antigen; CA19-9, carbohydrate antigen 19-9.

**Table 2:** Risk factors of early recurrence.

	Univariate			Multivariate		
	OR	(95% CI)	*p*-Value	OR	(95% CI)	*p*-Value
**Age**						
≤75	1.00					
>75	1.45	(0.83–2.53)	0.187			
**Sex**						
Female	1.00					
Male	1.28	(0.70–2.34)	0.418			
**BMI**						
<18.5	1.00			1.00		
18.5–24	0.54	(0.20–1.44)	0.219	0.89	(0.24–3.37)	0.864
≥24	0.36	(0.13–0.98)	0.045	0.59	(0.15–2.36)	0.461
**ECOG Performance Status**						
0–1	1.00			1.00		
>1	1.74	(0.99–3.06)	0.054	1.23	(0.51–2.97)	0.639
**aCCI**						
0–3	1.00					
>3	1.33	(0.74–2.41)	0.342			
**Location**						
Proximal	1.00					
Non-proximal	1.04	(0.54–1.98)	0.917			
**Size**						
≤4	1.00			1.00		
>4	3.12	(1.67–5.82)	<0.001	2.04	(0.84–5.00)	0.117
**Differentiation**						
Well to moderate	1.00					
Poor differentiation	1.71	(0.74–3.94)	0.211			
***Helicobacter pylori* infection**						
No	1.00					
Yes	1.01	(0.48–2.14)	0.979			
**Signet ring feature**						
No	1.00					
Yes	1.32	(0.72–2.42)	0.368			
**Gastrectomy type**						
Subtotal gastrectomy	1.00			1.00		
Total gastrectomy	2.31	(1.30–4.08)	0.004	2.04	(0.86–4.84)	0.106
**Lymphadenectomy type**						
<D2 dissection	1.00					
D2 dissection	0.68	(0.38–1.23)	0.206			
**T Stage**						
T 1–2	1.00					
T 3–4	1.90	(0.79–4.57)	0.153			
**N Stage**						
N 0–1	1.00			1.00		
N 2–3	3.44	(1.84–6.41)	<0.001	1.38	(0.50–3.84)	0.534
**Lymph nodes ratio**						
≤0.17	1.00			1.00		
>0.17	6.25	(3.41–11.45)	<0.001	5.30	(2.07–13.53)	<0.001
**Lymphovascular invasion**						
No	1.00			1.00		
Yes	2.81	(1.29–6.11)	0.009	1.05	(0.36–3.06)	0.926
**Perineural invasion**						
No	1.00			1.00		
Yes	2.22	(1.23–3.99)	0.008	1.96	(0.83–4.62)	0.122
**Margin**						
Negative	1.00			1.00		
Positive	4.57	(1.47–14.17)	0.008	1.83	(0.29–11.79)	0.523
**Adjuvant chemotherapy**						
No	1.00			1.00		
Yes	0.55	(0.30–1.00)	0.049	0.43	(0.16–1.12)	0.08
**CEA level**						
Normal	1.00			1.00		
Elevated (≥5 U/mL)	3.86	(1.92–7.78)	<0.001	2.02	(0.78–5.20)	0.145
**CA19–9 level**						
Normal	1.00			1.00		
Elevated (≥34 U/mL)	3.06	(1.50–6.23)	0.002	2.05	(0.82–5.08)	0.123
**Neutrophil-to-lymphocyte ratio**						
≤2.7	1.00					
>2.7	1.57	(0.90–2.76)	0.114			
**Platelet-to-lymphocyte ratio**						
≤164	1.00					
>164	1.31	(0.75–2.30)	0.341			
**Lymphocyte-to-monocyte ratio**						
≤3.1	1.00					
>3.1	0.65	(0.37–1.15)	0.138			
**Prognostic Nutritional index**						
≤45	1.00					
>45	1.11	(0.64–1.93)	0.717			
**Pan-immune inflammation value**						
≤305	1.00					
>305	1.31	(0.75–2.30)	0.341			

aCCI, age-adjusted Charlson comorbidity index; BMI, body mass index; ECOG, Eastern Cooperative Oncology Group; CEA, carcinoembryonic antigen; CA19-9, carbohydrate antigen 19-9.

## Data Availability

The datasets analyzed in this study can be obtained from the corresponding author upon reasonable request.

## Supplementary Materials

The following supporting information can be downloaded at https://www.mdpi.com/article/10.3390/jcm14186609/s1: Table S1. Regimens of adjuvant chemotherapy; Table S2. Hematologic side effects of chemotherapy (N = 169); Figure S1: Kaplan–Meier curves showing (a) disease-free survival (DFS) and (b) overall survival (OS) in patients aged ≥65 years with stage II/III resected gastric cancer, comparing those who underwent D1 versus D2 lymphadenectomy. DFS, disease-free survival; OS, overall survival.

## References

[1] Bray F., Laversanne M., Sung H., Ferlay J., Siegel R.L., Soerjomataram I., Jemal A. (2024). Global cancer statistics 2022: GLOBOCAN estimates of incidence and mortality worldwide for 36 cancers in 185 countries. CA Cancer J. Clin..

[2] Shin W.S., Xie F., Chen B., Yu P., Yu J., To K.F., Kang W. (2023). Updated Epidemiology of Gastric Cancer in Asia: Decreased Incidence but Still a Big Challenge. Cancers.

[3] Chang J.S., Kuo S.-H., Chu P.-Y., Shan Y.-S., Tsai C.-R., Tsai H.-J., Chen L.-T. (2019). The Epidemiology of Gastric Cancers in the Era of Helicobacter pylori Eradication: A Nationwide Cancer Registry-Based Study in Taiwan. Cancer Epidemiol. Biomark. Prev..

[4] del Arco C.D., Medina L.O., Muñoz L.E., Roldán E.M., Heras S.G.G.d.L., Aceñero M.J.F. (2023). Impact of Age at Diagnosis on Clinicopathological Features, Prognosis, and Management of Gastric Cancer: A Retrospective Single-Center Experience from Spain. Cancers.

[5] Ciesielski M., Kruszewski W.J., Szajewski M., Walczak J., Spychalska N., Szefel J., Zieliński J. (2019). Extremely High Mortality Rate after a Successful Gastrectomy for Cancer in Older Adults. J. Gastric Cancer.

[6] Japanese Gastric Cancer Association (2023). Japanese Gastric Cancer Treatment Guidelines 2021 (6th edition). Gastric Cancer.

[7] Shitara K., Fleitas T., Kawakami H., Curigliano G., Narita Y., Wang F., Wardhani S., Basade M., Rha S., Zamaniah W.W. (2024). Pan-Asian adapted ESMO Clinical Practice Guidelines for the diagnosis, treatment and follow-up of patients with gastric cancer. ESMO Open.

[8] Cuschieri A., Weeden S., Fielding J., Bancewicz J., Craven J., Joypaul V., Sydes M., Fayers P. (1999). Patient survival after D1 and D2 resections for gastric cancer: Long-term results of the MRC randomized surgical trial. Surgical Co-operative Group. Br. J. Cancer.

[9] Sasako M., Sakuramoto S., Katai H., Kinoshita T., Furukawa H., Yamaguchi T., Nashimoto A., Fujii M., Nakajima T., Ohashi Y. (2011). Five-year outcomes of a randomized phase III trial comparing adjuvant chemotherapy with S-1 versus surgery alone in stage II or III gastric cancer. J. Clin. Oncol..

[10] Liu D., Lu M., Li J., Yang Z., Feng Q., Zhou M., Zhang Z., Shen L. (2016). The patterns and timing of recurrence after curative resection for gastric cancer in China. World J. Surg. Oncol..

[11] Xu J., Shen L., Shui Y., Yu W., Guo Q., Yu R., Wu Y., Wei Q. (2020). Patterns of recurrence after curative D2 resection for gastric cancer: Implications for postoperative radiotherapy. Cancer Med..

[12] Von Elm E., Altman D.G., Egger M., Pocock S.J., Gøtzsche P.C., Vandenbroucke J.P. (2007). The Strengthening the Reporting of Observational Studies in Epidemiology (STROBE) statement: Guidelines for reporting observational studies. Ann. Intern. Med..

[13] Bouliaris K., Rachiotis G., Diamantis A., Christodoulidis G., Polychronopoulou E., Tepetes K. (2017). Lymph node ratio as a prognostic factor in gastric cancer patients following D1 resection. Comparison with the current TNM staging system. Eur. J. Surg. Oncol..

[14] Sun J., Chen X., Gao P., Song Y., Huang X., Yang Y., Zhao J., Ma B., Gao X., Wang Z. (2016). Can the Neutrophil to Lymphocyte Ratio Be Used to Determine Gastric Cancer Treatment Outcomes? A Systematic Review and Meta-Analysis. Dis. Markers.

[15] Cao W., Yao X., Cen D., Zhi Y., Zhu N., Xu L. (2020). The prognostic role of platelet-to-lymphocyte ratio on overall survival in gastric cancer: A systematic review and meta-analysis. BMC Gastroenterol..

[16] Ma J.Y., Liu Q. (2018). Clinicopathological and prognostic significance of lymphocyte to monocyte ratio in patients with gastric cancer: A meta-analysis. Int. J. Surg..

[17] Yu D., Liu J., Meng C., Liu B., Liao J. (2024). Pan-immune-inflammation value as a novel prognostic biomarker for digestive system cancers: A meta-analysis. World J. Surg. Oncol..

[18] Xishan Z., Ye Z., Feiyan M., Liang X., Shikai W. (2020). The role of prognostic nutritional index for clinical outcomes of gastric cancer after total gastrectomy. Sci. Rep..

[19] Kang W.M., Meng Q.B., Yu J.C., Ma Z.Q., Li Z.T. (2015). Factors associated with early recurrence after curative surgery for gastric cancer. World J. Gastroenterol..

[20] Qiu W.-W., Chen Q.-Y., Zheng W.-Z., He Q.-C., Huang Z.-N., Xie J.-W., Wang J.-B., Lin J.-X., Lu J., Cao L.-L. (2022). Postoperative follow-up for gastric cancer needs to be individualized according to age, tumour recurrence pattern, and recurrence time. Eur. J. Surg. Oncol..

[21] Wakatsuki K., Matsumoto S., Migita K., Kunishige T., Nakade H., Miyao S., Sho M. (2020). Risk Factors and Risk Scores for Predicting Early Recurrence After Curative Gastrectomy in Patients with Stage III Gastric Cancer. J. Gastrointest. Surg..

[22] Yagi S., Kumagai K., Nunobe S., Ishizuka N., Yamaguchi T., Imai Y., Tsuda M., Haruta S., Fukunaga H., Yamada T. (2024). Risk factors for early recurrence after radical gastrectomy followed by adjuvant chemotherapy for stage II or III gastric cancer: A multicenter, retrospective study. Jpn. J. Clin. Oncol..

[23] Ma M., Xiao H., Li L., Yin X., Zhou H., Quan H., Ouyang Y., Huang G., Li X., Xiao H. (2019). Development and validation of a prognostic nomogram for predicting early recurrence after curative resection of stage II/III gastric cancer. World J. Surg. Oncol..

[24] Supsamutchai C., Wilasrusmee C., Jirasiritham J., Rakchob T., Phosuwan S., Chatmongkonwat T., Choikrua P., Thampongsa T. (2020). Recurrence outcome of lymph node ratio in gastric cancer after underwent curative resection: A retrospective cohort study. Ann. Med. Surg..

[25] Park J., Jeon C.H., Kim S.J., Seo H.S., Song K.Y., Lee H.H. (2021). A Novel Approach for Gastric Cancer Staging in Elderly Patients Based on the Lymph Node Ratio. J. Gastric Cancer.

[26] Komatsu S., Ichikawa D., Nishimura M., Kosuga T., Okamoto K., Konishi H., Shiozaki A., Fujiwara H., Otsuji E. (2017). Evaluation of prognostic value and stage migration effect using positive lymph node ratio in gastric cancer. Eur. J. Surg. Oncol..

[27] Ke B., Song X.N., Liu N., Zhang R.P., Wang C.L., Liang H. (2014). Prognostic value of the lymph node ratio in stage III gastric cancer patients undergoing radical resection. PLoS ONE.

[28] Aoyama T., Komori K., Tamagawa A., Nakazano M., Hara K., Hashimoto I., Tamagawa H., Segami K., Maezawa Y., Kano K. (2022). Clinical Influence of the Lymph Node Ratio on Lymph Node Metastasis-positive Gastric Cancer Patients Who Receive Curative Treatment. In Vivo.

[29] Ergenc M., Uprak T.K., Akin M.I., Hekimoglu E.E., Celikel C.A., Yegen C. (2023). Prognostic significance of metastatic lymph node ratio in gastric cancer: A Western-center analysis. BMC Surg..

[30] Nelen S.D., van Steenbergen L.N., Dassen A.E., van der Wurff A.A., Lemmens V.E., Bosscha K. (2013). The lymph node ratio as a prognostic factor for gastric cancer. Acta Oncol..

[31] Seo H.S., Jung Y.J., Kim J.H., Park C.H., Lee H.H. (2018). Necessity of D2 lymph node dissection in older patients >/=80years with gastric cancer. J. Geriatr. Oncol..

[32] Sakaguchi M., Hosogi H., Kanaya S. (2022). Is D2 laparoscopic gastrectomy essential for elderly patients with advanced gastric cancer? A propensity score matched analysis. J. Gastrointest. Oncol..

[33] Degiuli M., Reddavid R., Tomatis M., Ponti A., Morino M., Sasako M., Rebecchi F., Garino M., Vigano L., Scaglione D. (2021). D2 dissection improves disease-specific survival in advanced gastric cancer patients: 15-year follow-up results of the Italian Gastric Cancer Study Group D1 versus D2 randomised controlled trial. Eur. J. Cancer.

[34] Shih Y.-H., Lin H.-C., Liao P.-W., Chou C.-W., Lin C.-H., Hsu C.-Y., Teng C.-L.J., Wu F.-H., Luo S.-C., Kao S.-H. (2023). The efficacy of adjuvant chemotherapy for older adults with stage II/III gastric cancer: A retrospective cohort study. BMC Cancer.

[35] Johnson M. (2012). Chemotherapy treatment decision making by professionals and older patients with cancer: A narrative review of the literature. Eur. J. Cancer Care.

[36] Kim H.S., Kim J.H., Kim J.W., Kim B.C. (2016). Chemotherapy in Elderly Patients with Gastric Cancer. J. Cancer.

[37] Lian L., Xia Y.-Y., Zhou C., Shen X.-M., Li X.-L., Han S.-G., Zheng Y., Mao Z.-Q., Gong F.-R., Wu M.-Y. (2015). Application of platelet/lymphocyte and neutrophil/lymphocyte ratios in early diagnosis and prognostic prediction in patients with resectable gastric cancer. Cancer Biomark..

[38] Deng Q., He B., Liu X., Yue J., Ying H., Pan Y., Sun H., Chen J., Wang F., Gao T. (2015). Prognostic value of pre-operative inflammatory response biomarkers in gastric cancer patients and the construction of a predictive model. J. Transl. Med..

[39] Caglar R. (2023). The relationship of different preoperative inflammatory markers with the prognosis of gastric carcinoma. Asian J. Surg..

[40] Eo W.K., Chang H.J., Suh J., Ahn J., Shin J., Hur J.-Y., Kim G.Y., Lee S., Park S., Lee S. (2015). The Prognostic Nutritional Index Predicts Survival and Identifies Aggressiveness of Gastric Cancer. Nutr. Cancer.

[41] Yang Y., Gao P., Song Y., Sun J., Chen X., Zhao J., Ma B., Wang Z. (2016). The prognostic nutritional index is a predictive indicator of prognosis and postoperative complications in gastric cancer: A meta-analysis. Eur. J. Surg. Oncol..

[42] Wang H.X., Wang C.C., Yang W., Gao L.L., Yu S.Q. (2018). Prognostic value of preoperative prognostic nutritional index in stage III gastric cancer after curative resection: A retrospective cohort study. Asia Pac. J. Clin. Nutr..

[43] Fest J., Ruiter R., Ikram M.A., Voortman T., van Eijck C.H.J., Stricker B.H. (2018). Reference Values for White Blood-Cell-Based Inflammatory Markers in the Rotterdam Study: A Population-Based Prospective Cohort Study. Sci. Rep..

[44] Stephenson S.S., Kravchenko G., Korycka-Błoch R., Kostka T., Sołtysik B.K. (2024). How Immunonutritional Markers Are Associated with Age, Sex, Body Mass Index and the Most Common Chronic Diseases in the Hospitalized Geriatric Population—A Cross-Sectional Study. Nutrients.

[45] Capurso C., Lo Buglio A., Bellanti F., Vendemiale G. (2024). Prognostic Nutritional Index and Instant Nutritional Assessment Are Associated with Clinical Outcomes in a Geriatric Cohort of Acutely Inpatients. Nutrients.

[46] Shih Y.H., Tsai Y.C., Lin H.C., Hsu C.Y. (2024). 167P The factors associated with early recurrence in older patients with stage II/III gastric cancer: A retrospective cohort study. Ann. Oncol..

