# Supplementary material for: Lymph Node Ratio as a Risk Factor for Early Recurrence in Older Patients with Stage II/III Gastric Cancer: A Retrospective Study
Source: J Clin Med. 2025 Sep 19;14(18):6609. doi: 10.3390/jcm14186609 (PMC12470672; doi:10.3390/jcm14186609)
Supplement: Supplementary file 1 [file jcm-14-06609-s001.zip › jcm-3843911-supplementary.pdf]

Table S1. Regimens of adjuvant chemotherapy

**Chemotherapy Regimens, n (%)**		N = 169
Single-agent regimen, n=130 (76.9%)	Tegafur-uracil (UFT)	56 (33.1)
	Tegafur-gimeracil-oteracil (S-1)	64 (37.9)
	5-Flurouracil	10 (5.9)
Combination regimen, n=39 (23.1%)	Capecitabine + Oxaliplatin	31 (18.3)
	Oxaliplatin + 5-Flurouracil	3 (1.8)
	Cisplatin + 5-Flurouracil	3 (1.8)
	Docetaxel + Tegafur-gimeracil-oteracil (S-1)	1 (0.6)
	Paclitaxel + 5-Flurouracil	1 (0.6)

Table S2. Hematologic side effects of chemotherapy (N=169)

	All grades		Grade 3/4	
**Leukopenia, n (%)**	51	(30.2)	1	(0.6)
**Neutropenia, n (%)**	37	(21.9)	4	(2.4)
**Anemia, n (%)**	134	(79.3)	5	(3.0)
**Thrombocytopenia, n (%)**	40	(23.7)	1	(0.6)
**Febrile neutropenia, n (%)**	2	(1.2)	0	(0.0)

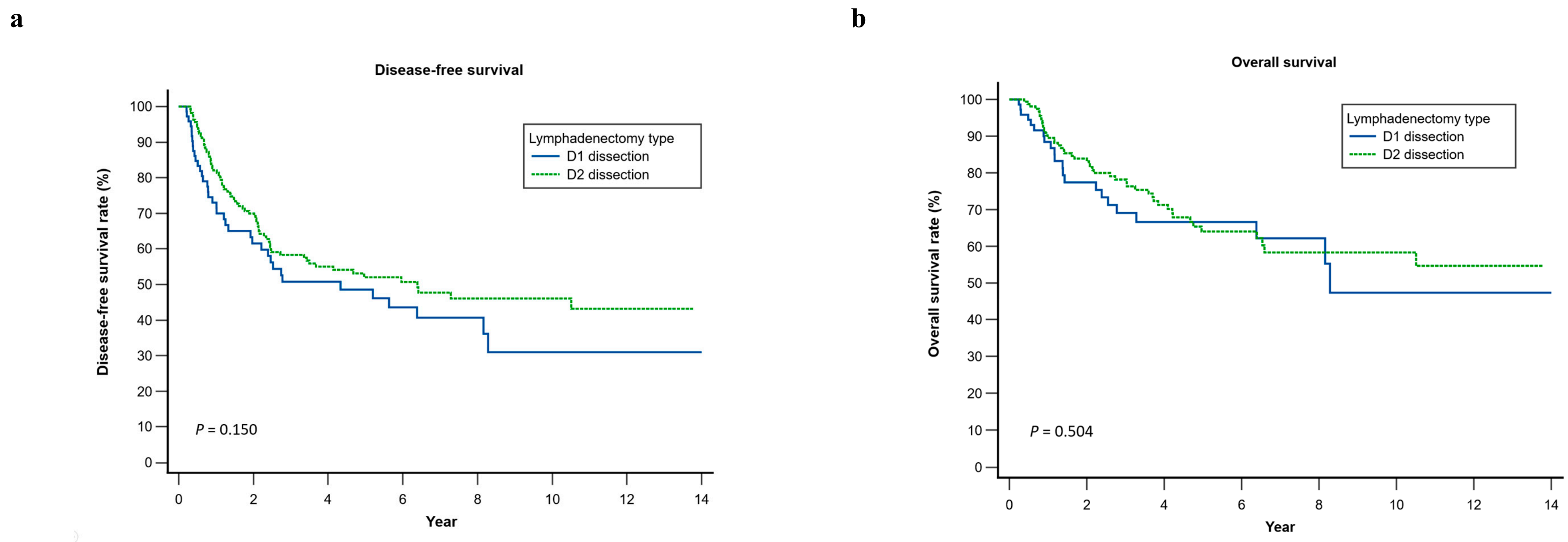

Figure S1

Kaplan–Meier curves showing (a) disease-free survival (DFS) and (b) overall survival (OS) in patients aged ≥65 years with stage II/III resected gastric cancer, comparing those who underwent D1 versus D2 lymphadenectomy.

DFS, disease-free survival; OS, overall survival.
